# Intractable hepatic hydrothorax eliminated by thoraco-peritoneal connection: A case report

**DOI:** 10.1097/MD.0000000000050685

**Published:** 2026-09-18

**Authors:** Qing Lang, Xiaoyuan Ben, Chengkang Wang, Jiebing Zhang, Xinguo Wang

**Affiliations:** a Department of Hepatology, Dazhou Central Hospital, Da Zhou, Sichuan, People’s Republic of China; b Department of Infectious, The Fourth Affiliated Hospital Zhejiang University School of Medicine, Yi Wu, Zhejiang, People’s Republic of China.

**Keywords:** case, decompensated cirrhosis, hepatic hydrothorax, prognosis, thoraco-peritoneal connection

## Abstract

**Rationale:**

Hepatic hydrothorax occurs in patients with decompensated cirrhosis and has a significantly adverse prognosis. However, several treatments, including indwelling pleural catheters, trans jugular intrahepatic systemic shunts, and automatic low-flow ascites pumps (Alfa pumps), have been utilized to relieve pleural effusion, but these methods often cause severe complications. We report the first case of using thoraco-peritoneal connection to manage hepatic hydrothorax in a cirrhotic patient without an unfavorable outcome.

**Patient concerns:**

A 55-year-old yellow-skinned female patient with alcoholic cirrhosis suffering from hepatic hydrothorax was admitted to the hospital. She presented with recurrent yellowish complexion, anorexia, chest tightness, and shortness of breath.

**Diagnoses:**

The definitive diagnosis encompassed liver failure, alcoholic cirrhosis in a decompensated state, esophageal varices, portal hypertension, ascites, hepatic hydrothorax, hypersplenism, cholecystolithiasis accompanied by cholecystitis, pulmonary nodules, and coronary atherosclerosis.

**Interventions:**

A thoracic drainage catheter and an abdominal puncture indwelling needle were connected to allow the pleural effusion to continuously flow into the abdominal cavity.

**Outcomes:**

The patient showed rapid improvement in nutritional status, urine output, and a decrease in pleural effusion. Subsequently, pleural effusion did not increase, and the connection was removed 1 month later. Hydrothorax and ascites were examined by color Doppler ultrasound every 2 months. Liver function and coagulation function continued to improve. The patient resumed normal daily activities after 6 months.

**Lessons:**

The management of hepatic hydrothorax remains an area requiring further investigation. Thoraco - peritoneal connection might represent a medical strategy for the management of hepatic hydrothorax in cirrhotic patients with a favorable safety profile.

## 1. Introduction

Hepatic hydrothorax is observed in 5% to 15% of patients with decompensated cirrhosis.^[1]^ Significantly, patients with decompensated liver failure accompanied by hepatic hydrothorax exhibit a 1-year mortality rate of up to 57%,^[2]^ Several treatment options exist for relieving pleural effusion, such as an indwelling pleural catheter or an automatic low-flow ascites pump (Alfapump).^[3]^ Although these methods yield some therapeutic outcomes, they are associated with certain adverse effects. Herein, we report a case of intractable hepatic hydrothorax alleviated through thoraco-peritoneal connection. This case underscores the potential of this novel strategy and the wider applicability of thoraco-peritoneal connection in patients with intractable hepatic hydrothorax.

## 2. Case presentation

A 55-year-old female patient presented with a yellowish complexion and anorexia for 5 years prior to being diagnosed with alcoholic cirrhosis by gastroscopy at another comprehensive hospital. One year after diagnosis, the patient was admitted to the emergency department of our hospital, presenting with paroxysmal and exacerbating intractable pain in the right upper abdomen with no identifiable trigger. Computed tomography angiography (CTA) of the mesenteric arteries and veins revealed local distal torsion of the superior mesenteric artery. Meanwhile, contrast-enhanced computed tomography (CT) of the whole abdomen indicated a series of manifestations, including gallbladder stones, cholecystitis, fat stranding in the peritoneal spaces, and the presence of fluid collection in the abdominal and pelvic cavities. Moreover, cirrhosis of the liver and splenomegaly were noted. Subsequently, diuretic therapy and intermittent abdominal drainage were carried out in the general surgery ward with the drainage of ascites at a rate of 1000ml per day for 7 days before discharge with a small quantity of drainage (<400ml). During the subsequent 2-year follow-up period, the patient was admitted to our department on 8 occasions for recurrent abdominal distension, anorexia, and hepatic decompensation. She subsequently developed chest tightness and shortness of breath on February 17, 2024, due to a large right-side pleural effusion with minimal ascites.

The patient had a medical history of lumbar fracture that occurred 2 years ago, which was treated with percutaneous vertebroplasty with balloon kyphoplasty. One year ago, the patient underwent splenic artery embolization for hypersplenism. There was a history of gallbladder stones, cholecystitis, pulmonary nodules, and coronary and aortic atherosclerosis. The patient reported no history of hypertension, diabetes, chronic bronchitis, or other chronic diseases. The patient also reported no history of major infectious diseases such as tuberculosis. There was a history of blood transfusion but she denied any poisoning or drug allergy.

The patient, who was born and resided in China, was illiterate and widowed. She exhibited no allergies to medications or food and reported no history of cigarette smoking. She had consumed approximately 60g of alcohol daily for 30 years but had abstained for 3 years since diagnosis of alcoholic cirrhosis. Her diet was regular. There was no significant family history of diabetes mellitus, hypertension, or cancer. She lived with her son, who provided primary care.

On admission, vital signs were stable. Scleral and skin icterus were noted. No palmar erythema or spider angiomas were observed. Auscultation revealed diminished breath sounds and dullness to percussion over the right hemithorax. Cardiac and left lung auscultation were unremarkable. The abdomen was soft, non-tender, and without rebound. Mild bilateral lower extremity edema was present.

Serum total bilirubin was 64.4μmol/L (normal range < 21μmol/L), with direct bilirubin at 32.4μmol/L (normal range < 17μmol/L). The patient had anemia with a hemoglobin of 72 g/L. Platelet count was 65 × 10^9^/L (normal range 100–300 × 10^9^/L). White blood cell count was 7.8 × 10^9^/L. Coagulation parameters were markedly abnormal: prothrombin time was 17.3 seconds (normal range 9.8–12.3 seconds), partial thromboplastin time was 33.9 seconds (normal range 23.9–33.5 seconds), and international normalized ratio was 1.53 (normal range 0.9–1.1). Initial serum glucose was within the normal range at 4mmol/L (normal range 2.5–7.0mmol/L). Liver enzymes were within normal limits: aspartate aminotransferase 41 IU/L (13–40) and alanine aminotransferase 37 IU/L (7–45). Serum ammonia was normal at 64μmol/L, and serum albumin was reduced to 30g/L (40–55). Renal function tests showed: serum creatinine was 66μmol/L (normal range 41–73μmol/L), blood urea nitrogen was 7.77 mmol/L (normal range 2.6–7.5mmol/L), and uric acid was 573.7μmol/L (normal range 142–416μmol/L). Infectious workup was negative.

**Figure 1.**
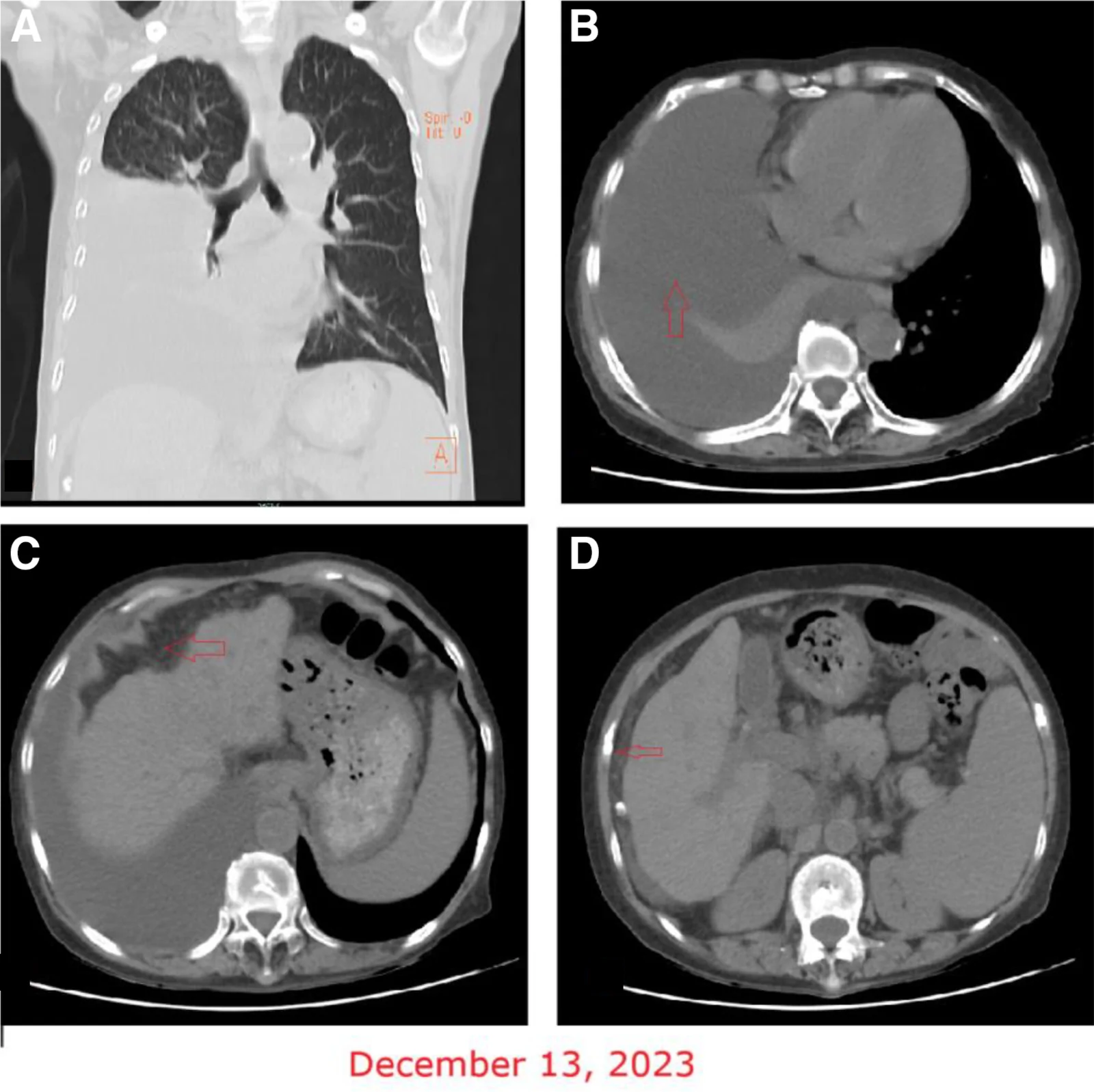
Pulmonary CT imaging findings of hepatic pleural effusion. (A): In the right thoracic cavity, there was large right pleural effusion with passive atelectasis, accompanied by inferior displacement of the right diaphragm and superior displacement of the left diaphragm.(B): The right lower lobe was fully compressed, and there was no ventilation within the lung.(C): The right diaphragmatic folds exhibited variable thicknesses, and some formed a 1-way valve-like structure connecting the abdominal cavity to the thoracic cavity. The liver surface was irregular, with poor adhesion to the diaphragm. (D): There was no notable ascites in the abdominal cavity, and only minimal perihepatic ascites was detected. CT = computed tomography.

Lung computed tomography (CT) revealed a substantial right pleural effusion with adjacent pulmonary atelectasis. Multiple small nodules in the right lung and ground-glass opacities in the left upper lobe were seen. Atherosclerotic calcifications were observed in the coronary arteries and the aorta. Cirrhosis, along with splenomegaly and ascites, was present, and peritoneal fat stranding was noted (Fig. 1).

Based on the above medical history, various laboratory tests, and examination, the patient was diagnosed with liver failure, alcoholic cirrhosis in a decompensated state, esophageal varices, portal hypertension, ascites, hepatic hydrothorax, hypersplenism, cholecystolithiasis accompanied by cholecystitis, pulmonary nodules, and coronary atherosclerosis.

Given that the patient’s main symptoms (anorexia, chest tightness, and dyspnea) were caused by massive right-sided pleural effusion compressing the lung, the primary goal was to reduce the pleural fluid volume and facilitate its drainage. The underlying mechanism was a unidirectional diaphragmatic defect, which allowed ascites to flow preferentially into the pleural space. Our treatment strategy aimed to reverse this flow by draining the pleural effusion back into the peritoneal cavity.

Before thoracentesis, the patient was treated with to rasemide in combination with spironolactone for diuretic therapy, silymarin for liver protection and enzyme reduction, and ademetionine butyric disulfonate for cholangitis.

**Figure 2.**
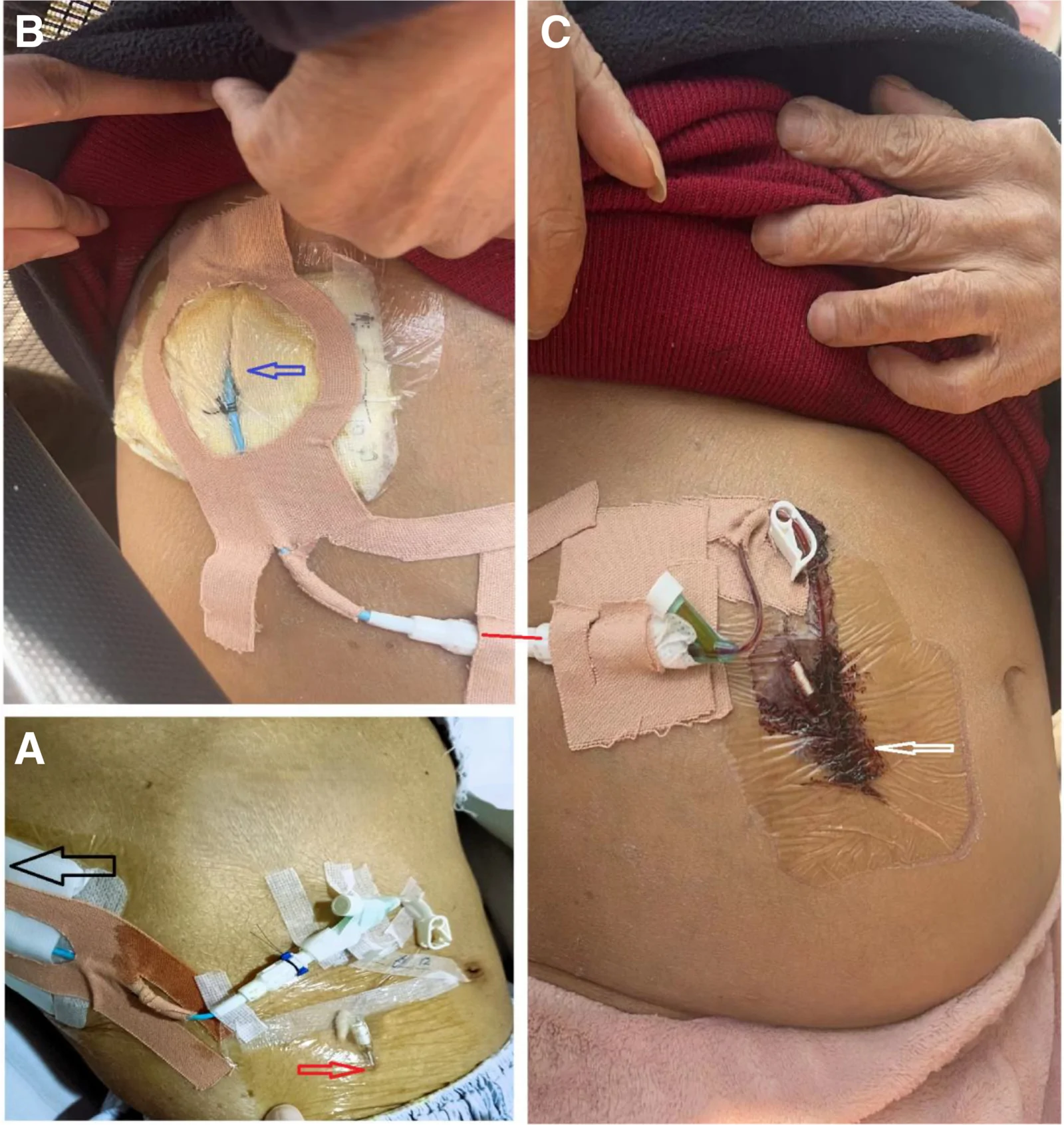
Alteration after thoracoperitoneal connection. Image A illustrated the initial puncture and connection. There was no ascites in the abdominal cavity; ascites only presented in the thoracic cavity. Images (B) and (C) were taken 3 days after the connection, showing abdominal distension. However, the patient’s skin color improved significantly, indicating restored blood perfusion, and jaundice was also markedly alleviated. Image (B) depicted the puncture site of the thoracic drainage tube, and Image (C) showed the puncture site of the abdominal indwelling needle, with a small amount of abdominal wall bleeding into the indwelling needle catheter. (A): Initial state of puncture and connection (B): Variations of Puncture Site in thoracentesis 3 days after the connection (C): Variations in abdominal paracentesis site 3 days after the connection.

In the pleural effusion catheterization process, the patient was seated with back support. The puncture site was selected using ultrasound guidance and confirmed by percussion dullness. After disinfection and local anesthesia, lidocaine was infiltrated layer by layer using a 5-mL syringe until yellow pleural fluid was aspirated. Then, a puncture needle was inserted along the same tract, a guidewire was advanced, and a drainage catheter was placed. After the procedure, the site was disinfected and dressed (Figure 2A, red arrow).

Four days later, despite daily drainage of 1000 mL, the patient’s chest tightness persisted. Abdominal CT showed a fluid collection about 10 cm to the right of the umbilicus, and dullness was elicited on percussion. After confirming fluid, the indwelling needle was advanced 1 cm further and secured (Figure 2A: Black arrow, B: Blue arrow). Notably, the pleural drainage fluid transiently turned reddish during abdominal paracentesis.

The patient’s chest drainage tube and abdominal indwelling needle were connected via a connecting tube (Fig. 2). On the first day after connection, the patient had increased urine output and mild abdominal distension, but chest tightness was markedly relieved. By day 3, chest tightness had resolved, but ascites increased significantly (Figure 2C); her mental status improved notably. Hemoglobin remained stable.

On day 4 after thoraco-peritoneal connection, the patient’s vital signs remained stable, accompanied by a notable alleviation of chest tightness and an augmentation in urine output. Both puncture sites were dry without exudate, and no fresh blood was seen in the abdominal catheter. The patient was discharged with advice to maintain a normal diet, abstain from alcohol, and continue oral to rasemide and spironolactone. She was instructed to keep puncture sites dry and to contact the physician if abdominal pain occurred. One month after discharge, while still at home with the drainage system, no pleural or peritoneal effusion was observed on ultrasound, and the system was removed.

During follow-up, renal and liver function gradually improved. Thereafter, the patient was reevaluated every 2 months. Pleural and peritoneal effusions remained stable; serum protein and bilirubin normalized, except for a mild fluctuation at month 5. She denied chest tightness or dyspnea, despite a small residual left pleural effusion. Assessment attributed this to inadequate protein intake, and increased dietary protein was advised. One month later, these values returned to normal. At the most recent follow-up (8 months), pleural and peritoneal effusions remained stable without recurrence of large-volume effusion. Liver and renal function had essentially normalized, and quality of life was markedly improved compared with admission. She had resumed light physical activities and regular work. (Table 1, 2)

**Table 1:** Findings from color Doppler ultrasound in measuring the alterations of thoracoabdominal fluid subsequent to thoracoabdominal communication.

	2024.02.17	2024.04.11	2024.05.12	2024.07.26	2024.08.28	2024.10.18	2024.11.10	2025.01.08	2025.03.07
Ascites (cm)									
Perihepatic (cm)	1.46	0	0	0	0	0	0	0	0
Right lower quadrant (cm)	-	0	0	0	0	0	0	0	0
Pelvic cavity	1.96	0.86	0	0	0	1.55	0	0	0
Left lower abdomen (cm)	1.17	0	0	0	0	0	0	0	0
Pleural effusion									
Right thoracic cavity (cm)	7.66	1.76	1.64	1.41	0.98	1	0	0	0
Left thoracic cavity (cm)	0	0	2.34	0	0	1.61	0	0	0

The data in table 1 suggest that the volume of fluid in each cavity before thoracoabdominal connection mainly manifested as right-sided pleural effusion. During continuous observation subsequent to thoracoabdominal connection, the right pleural effusion gradually diminished and vanished after 6 months. Two instances of mild pleural effusion were sporadically observed in the left thoracic cavity, showing no association with the right pleural effusion or ascites. The ascites was also confined to a small quantity within the pelvic cavity.

**Table 2:** Changes in liver and kidney function after thoracic cavity connection.

	2024.02.17	2024.04.11	2024.05.12	2024.07.26	2024.08.28	2024.10.18	2024.11.10	2025.01.07	2025.03.07
PT (s)	16.6	15.4	14.3	16.7	15.7	17.8	16.5	15.7	16.6
PLT (×10^9/L)	55	39	49	37	44	39	48	58	54
TB (µmol/L)	52.9	52.3	60.6	66.5	49.5	46.5	41.5	33.3	37.1
DB (µmol/L)	27.7	23.6	26.6	29.6	19.7	20.2	17.4	13.5	14.2
ALT (U/L)	31	31	33	31	23	19	17	27	22
AST (U/L)	39	37	39	48	40	40	33	43	42
ALB (g/L)	26.6	33	34.5	37.1	36.9	35.3	38.3	38.9	40.4
GLB (g/L)	32.3	37	38.6	37.4	37	36.3	40.6	38.9	39.9
BUN (mmol/L)	7.77	6.15	5.19	4.48	6.87	4.83	5.6	6.47	5.97
CR (µmol/L)	66	65	64	75	63	61	57	61	63
UA (µmol/L)	472.9	729.4	558.8	696.6	517.7	677	541	522	594

Table 2 reveals a significant rebound in serum albumin levels and a considerable increase in serum uric acid levels 1 month after thoracoabdominal shunt. Liver and kidney functions gradually reverted to normal. Bilirubin and transaminase levels gradually decreased, while albumin and globulin levels gradually recovered, approaching the normal ranges within 6 months. The renal excretion of urea nitrogen increased gradually, whereas the excretion of uric acid lagged behind that of urea. Creatinine levels exhibited minimal fluctuations throughout the observation period with no statistically significant differences.

ALB = albumin, ALT = alanine aminotransferase, AST = aspartate aminotransferase, BUN = blood urea nitrogen, CR = creatinine, DB = direct bilirubin, GLB = globulin, PLT = platelet, PT = prothrombin time, TB = total bilirubin, UA = uric acid.

## 3. Discussion

Hepatic hydrothorax typically occurs in cirrhotic patients with portal hypertension and significant ascites.^[2,4]^ In our patient, creating a thoraco-peritoneal communication diverted pleural fluid into the peritoneal cavity, effectively interrupting the pathophysiological cycle. This intervention interrupted the key mechanism, converting a vicious cycle into a favorable clinical course.

The transition from uncomplicated ascites to refractory ascites is the fundamental event in hepatic hydrothorax. Uncomplicated ascites refers to any ascites that is not infected, not associated with renal failure, not refractory, and does not require specific treatment. The paradigm of uncomplicated ascites formation in liver cirrhosis is contingent upon portal hypertension, dysregulated splanchnic vasodilation, and effective hypovolemia. However, when the following mechanisms become the primary factors in the formation of ascites, the ascites turns into refractory ascites. Activation of neuro humoral systems promotes vasoconstriction and renal retention of sodium and water. Portal hypertension increases intestinal mucosal permeability and facilitates the translocation of pathogen-associated molecular patterns from the gut lumen. Hypoproteinemia persists if not corrected. Repeated diuretic use may reduce renal responsiveness. Ascites due to these mechanisms becomes refractory and recurs early.^[5]^

Treatment principles for hepatic hydrothorax are similar to those for refractory ascites, including sodium restriction, diuretics, and portal pressure reduction. However, these measures are often insufficient.^[6–9]^ Transjugular intrahepatic portosystemic shunt (TIPS), indwelling pleural catheters, and pleurodesis have been proposed; they can alleviate symptoms in 70 to 80% of cases.^[10–12]^However, pleurodesis carries infection rates of 5% to 35%, mortality of 0% to 3.2%, and an overall complication rate of 82%.^[13]^ The Alfa pump® may be suitable for non-loculated, non-sanguineous hydrothorax without empyema.^[14]^ However, it requires specialized equipment and patient tolerance.

The widely accepted mechanism is that ascitic fluid traverses minor defects in the diaphragm and enters the thoracic cavity.^[15]^ In our patient, CT showed right-sided predominance, consistent with the literature.^[2,16]^ The key factor is diaphragmatic injury at the liver bare area, likely due to cirrhosis. This injury acts as a unidirectional valve. In our patient, this was evidenced by the appearance of red blood cells in the pleural drainage fluid during abdominal paracentesis. Prehospital ultrasound showed dynamic changes: when ascites decreased, pleural effusion increased, confirming a communication between cavities. Although diaphragmatic defects are described, the exact mechanism of injury in cirrhosis is not well documented. The patient had massive ascites for 2 to 3 years before the procedure. We hypothesize that upward pressure from ascites combined with negative intrathoracic pressure caused diaphragmatic fiber tears, creating a unidirectional flow.

Our intervention reversed this gradient: pleural fluid drained into the peritoneal cavity due to the pressure difference. By connecting the pleural and peritoneal spaces, a hydrostatic gradient (with pleural pressure normally 4–6 cm H_2_O) drives fluid downwards; expiratory pressure further augments flow. Given that pleural pressure is negative, a height difference of >4 to 6 cm between puncture sites ensures unidirectional flow. This is analogous to underwater seal drainage, explaining the sustained clinical benefit.

Peritoneal absorption of ascites appeared enhanced after connection, as pleural volume decreased without a commensurate increase in ascites. A significant decrease in the volume of pleural effusion was observed, whereas the volume of abdominal fluid did not show a corresponding increase. This suggests an enhanced peritoneal absorption capacity for ascites, at least following the establishment of thoraco abdominal communication. This may be related to the return of protein-rich pleural fluid to the peritoneal cavity, raising plasma albumin levels.

The establishment of thoraco-peritoneal communication serves as a promising therapeutic strategy for carefully selected patients with refractory hepatic hydrothorax, presenting a minimally invasive alternative to more intricate surgical interventions. However, this is a single-case report; larger prospective studies are needed. Moreover, precise abdominal puncture is essential to avoid bowel injury; real-time ultrasound with 3-dimensional guidance may be helpful. Second, patient movement may cause shear stress on the needle, necessitating robust materials to prevent fracture. Third, minor transient bleeding at the puncture site is usually self-limiting and does not affect outcome.

## 4. Conclusion

In conclusion, this case provides preliminary evidence that thoraco-peritoneal connection may be a feasible and effective strategy for refractory hepatic hydrothorax, warranting further investigation.

## Author contributions

**Data curation:** Chengkang Wang, Jiebing Zhang.

**Investigation:** Chengkang Wang, Jiebing Zhang.

**Project administration:** Xinguo Wang.

**Writing – original draft:** Qing Lang.

**Writing – review & editing:** Xiaoyuan Ben.
